# Survival of MS2 and Φ6 viruses in droplets as a function of relative humidity, pH, and salt, protein, and surfactant concentrations

**DOI:** 10.1371/journal.pone.0243505

**Published:** 2020-12-08

**Authors:** Kaisen Lin, Chase R. Schulte, Linsey C. Marr

**Affiliations:** Department of Civil and Environmental Engineering, Virginia Tech, Blacksburg, Virginia, United States of America; Universidade de Aveiro, PORTUGAL

## Abstract

The survival of viruses in droplets is known to depend on droplets’ chemical composition, which may vary in respiratory fluid between individuals and over the course of disease. This relationship is also important for understanding the persistence of viruses in droplets generated from wastewater, freshwater, and seawater. We investigated the effects of salt (0, 1, and 35 g/L), protein (0, 100, and 1000 μg/mL), surfactant (0, 1, and 10 μg/mL), and droplet pH (4.0, 7.0, and 10.0) on the viability of viruses in 1-μL droplets pipetted onto polystyrene surfaces and exposed to 20%, 50%, and 80% relative humidity (RH) using a culture-based approach. Results showed that viability of MS2, a non-enveloped virus, was generally higher than that of Φ6, an enveloped virus, in droplets after 1 hour. The chemical composition of droplets greatly influenced virus viability. Specifically, the survival of MS2 was similar in droplets at different pH values, but the viability of Φ6 was significantly reduced in acidic and basic droplets compared to neutral ones. The presence of bovine serum albumin protected both MS2 and Φ6 from inactivation in droplets. The effects of sodium chloride and the surfactant sodium dodecyl sulfate varied by virus type and RH. Meanwhile, RH affected the viability of viruses as shown previously: viability was lowest at intermediate to high RH. The results demonstrate that the viability of viruses is determined by the chemical composition of carrier droplets, especially pH and protein content, and environmental factors. These findings emphasize the importance of understanding the chemical composition of carrier droplets in order to predict the persistence of viruses contained in them.

## Introduction

Pathogenic organisms, including bacteria, viruses, fungi, protozoa, and helminths, cause infections that are a leading cause of global morbidity and mortality [1]. In particular, viruses are responsible for diseases such as COVID-19, influenza, hepatitis, Ebola virus disease, and many cases of gastroenteritis. Some of these diseases rely on the spread of viruses in the environment, from infected hosts to susceptible hosts via aerosol, droplet, fomite, and/or fecal-oral routes. Successful transmission requires that the virus survive, or maintain its infectivity, while it is in the environment. Studies on the persistence of viruses in solutions, on material surfaces, and in the air have shown that survival varies by strain, composition of the surrounding media, and environmental factors [2–9].

**Fig 1:**
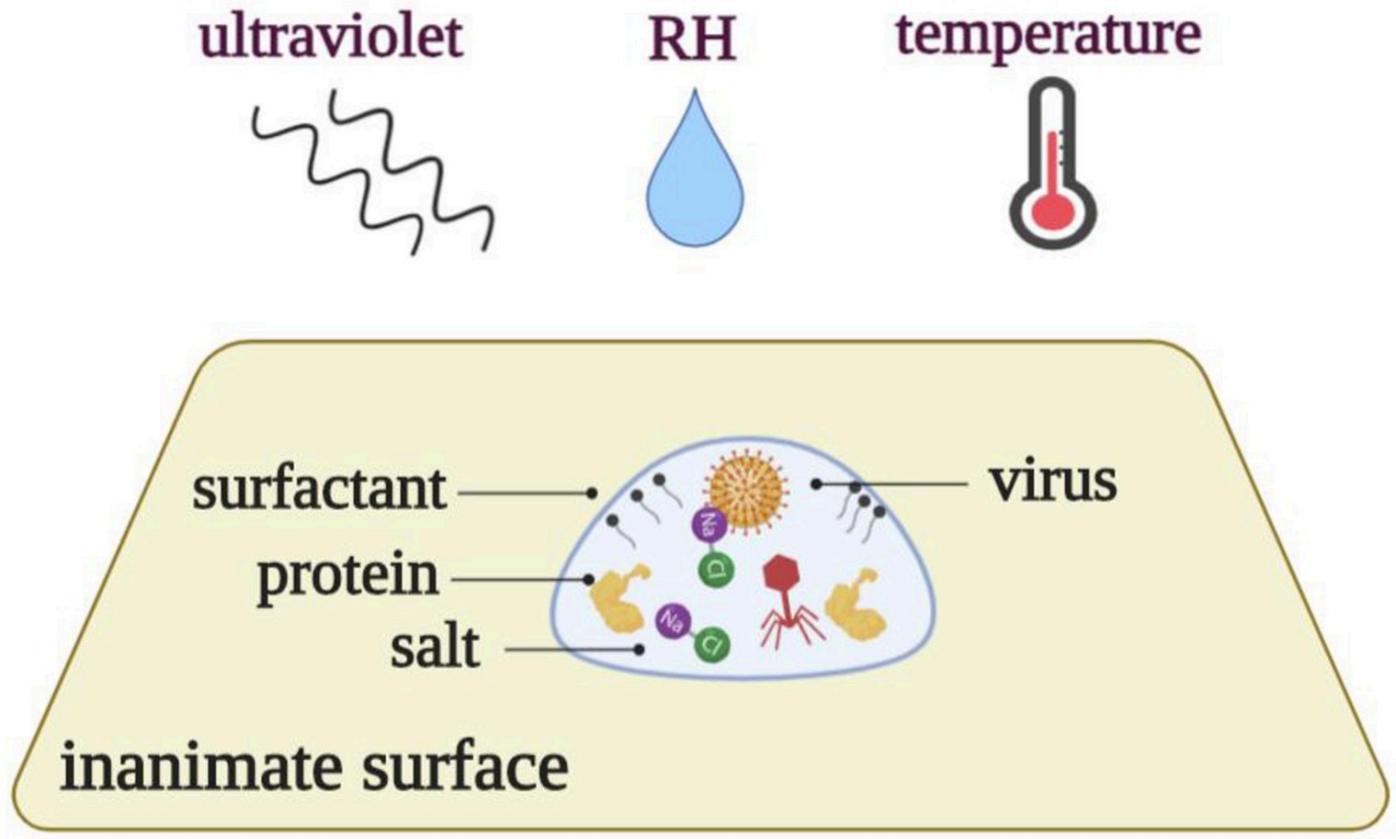
Factors that affect the survival of viruses in stationary droplets on surfaces.

Aerosol, droplet, and fomite transmission are important routes for the spread of many viral diseases, such as influenza and measles [10]. Viruses can be released from an infected individual or natural sources in aerosols (microscopic droplets small enough to remain airborne for minutes or more) and droplets (larger droplets that quickly deposit onto surfaces). Natural sources of viruses in aerosols and droplets include, for example, seawater [11] and wastewater systems [12]. Aerosols and droplets are complex systems due to their varying size, high surface-to-volume ratio, and spatially heterogeneous composition. Viruses that are immersed in aerosols and droplets experience a dynamic and highly variable microenvironment as aerosols and droplets evaporate and equilibrate with ambient environmental conditions, as illustrated in Fig 1; changes in the microenvironment may affect the viruses’ viability [13].

Aerosols and droplets emitted from different sources may have distinct chemical and physical properties. For example, fluids expelled by infected patients when they exhale, talk, cough, or sneeze contain high levels of proteins and are usually viscous [14], while sea spray aerosols contain more salts and organic compounds [15]. Because each chemical component might have a different effect on a virus, its survival in aerosols and droplets that are generated from different sources may vary [16].

Relatively few studies have investigated the effects of aerosol and droplet composition on the viability of viruses; these studies have focused mainly on the effects of salts and/or proteins. We have shown that high salt concentrations were associated with reduced viability of influenza virus in droplets [17]. In a separate study, we found that influenza virus better retained infectivity in droplets of culture medium that was supplemented with human epithelial bronchial (HBE) cell wash compared to the culture medium alone [18]. HBE cell wash also promoted the survival of the bacteriophage Φ6: it decayed ~2 log_10_ units in tryptic soy broth (TSB) after 1 h of exposure to 75% RH in aerosols, whereas it did not decay in HBE cell wash under the same conditions [18]. We suspect that proteins in HBE cell wash might have protected the viruses, but the exact reason for the observed difference remains unknown.

Prior work has focused almost exclusively on virus viability in culture media. However, viruses in the real world are not immersed in culture media; they are present in respiratory fluid, wastewater, freshwater, seawater, and other fluids. Given the complexity of environmental matrices, a more complete understanding of the effect of individual media components on the inactivation of viruses in aerosols and droplets is needed. In addition to salts and proteins, the levels of surfactants and pH vary substantially in environmentally and physiologically relevant fluids [19, 20]. Experiments are needed to disentangle the effects of different chemical components of the fluid on virus viability across the range of concentrations found in environmentally and physiologically relevant sources.

The viability of viruses in aerosols and droplets is also affected by environmental factors, such as temperature [21, 22], humidity [16–18, 23–27], and ultraviolet radiation [28–30]. Temperature and ultraviolet radiation primarily affect the viability of viruses by destroying their structural integrity [31]. Other studies have shown that the viability of viruses in both aerosols and droplets depends on relative humidity (RH) [18, 26, 27]. Our previous studies have reported a U-shaped pattern in virus viability as a function of RH in both aerosols and droplets; viruses survived well at RHs lower than 40% or near 100%, but their viability was reduced at intermediate RH [13, 18]. We have proposed that this relationship is mediated by changes in the physicochemical properties of aerosols and droplets as they evaporate to equilibrate with ambient RH [10, 32]. To our best knowledge, there have not been any studies that explore the interaction effects of media composition and relative humidity on virus survival in droplets.

In this study, we manipulated the concentrations of several common media components, including salt, protein, and surfactant, as well as pH, over environmentally and physiologically relevant ranges, while quantifying the viability of model viruses in droplets of the media. To gain insight into the interactions between media composition and RH on the viability of viruses in evaporating droplets, we exposed the virus-containing droplets to low, intermediate, and high RH levels for 1 h and evaluated the reduction in virus viability. Results from this study will provide information on the effects of specific media components on the decay of non-enveloped and enveloped viruses in droplets. We hope that addressing this question may enable advances in understanding the mechanisms of virus inactivation in the environment.

## Materials and methods

### Virus stock

Bacteriophage MS2 (ATCC 15597-B1), a model for non-enveloped RNA viruses that is widely used in environmental engineering studies [33–35], was propagated as previously described [12]. Briefly, *E*. *coli* (ATCC 15597) was inoculated in Miller’s Luria Broth (LB) medium (Fisher Scientific) and incubated overnight at 37°C. Fifty microliters of MS2 stock, 450 μL of *E*. *coli* liquid culture, and 4.5 mL of LB soft agar were mixed and overlaid on LB agar plates. Plates were incubated at 37°C for 24 h before the top layer of soft agar was collected in LB medium. The mixture of soft agar and LB medium was shaken at 100 rpm at 37°C for 2 h. The mixture was then centrifuged at 5000 rpm for 5 min, and the supernatant was filtered through a 0.22 μm cellulose acetate membrane to remove cells and debris. The filtrate was collected as virus stock and stored at 4°C.

Bacteriophage Φ6 (kindly provided by Dr. Paul Turner at Yale University), a model for enveloped RNA viruses including influenza virus [4, 35–37], was propagated using the same method described above, except that the bacterial host was *Pseudomonas syringae*, which grows at 25°C. Determined by plaque assay, the concentrations of virus stocks were 10^10^−10^11^ plaque-forming units (PFU)/mL.

### Preparation of virus suspension

For testing the effect of different components of media on virus viability, solutions containing various amounts of salt, protein, and surfactant were prepared. Specifically, a 100 g/L NaCl stock solution was prepared by adding 100 g of sodium chloride (Fisher Scientific) to ultrapure water (Barnstead Nanopure; Thermo) to a final volume of 1 L. Aliquots of the solution were then diluted with ultrapure water to produce working solutions with the concentrations shown in Table 1. Stock solutions of bovine serum albumin (BSA) (Sigma), a protein derived from cows, and sodium dodecyl sulfate (SDS) (Sigma), an anionic surfactant used in many cleaning and hygiene products, were prepared and diluted similarly as the NaCl stock solution.

**Table 1:** Chemicals used to make solutions and their concentrations in virus suspensions.

Component	Chemical(s) used	Stock solution concentration	Concentration/pH tested	Relevance
Salt	Sodium chloride	100 g/L	0, 1, 35 g/L	Salinity of seawater: 35 g/L
				Salinity of surface water: 0.01 to few g/L [38]
				Lung fluid: ~10 g/L [39]
Protein	Bovine serum albumin	100 mg/mL	0, 100, 1000 μg/mL	Respiratory fluid: 30–8500 μg/mL [25, 40–42]
Surfactant	Sodium dodecyl sulfate	100 μg/mL	0, 1, 10 μg/mL	Lung surfactant: up to 1000 μg/mL [19, 43]
				Surface water: less than 1 μg/mL [20]
pH	Hydrochloric acid, sodium hydroxide	NA	4.0, 7.0, 10.0	Ambient aerosol: 0–4 [44, 45]
				Inorganic aerosol: basic [46]
				Human respiratory fluid: pH-neutral [47]

NA indicates no stock solution with different pH values was prepared.

Virus suspensions at the targeted concentrations of salt, protein, and surfactant were prepared right before experiments were conducted. Virus stock and the working solutions were mixed at a ratio of 1:100. Specifically, 50 μL of virus stock was diluted with 4.95 mL of the working solution of interest in 15 mL centrifuge tubes and vortexed for 30 seconds. Virus stock and ultrapure water, which had an initial pH of 5.5, were mixed at the same ratio. The pH of the mixture was adjusted to 4.0, 7.0, or 10.0 with 0.1 M hydrochloric acid or 0.1 M sodium hydroxide and was measured using a pH meter (Orion Versa Star; Thermo). To avoid introducing excessive ions to the mixture, no pH buffer was added after the pH was adjusted to the target value in the mixture. The amount of ions introduced to the solutions to adjust the pH, shown in Table 1, was much lower than that used to make the 1 g/L NaCl working solution. Thus, the change in ionic strength due to pH adjustment should have a negligible effect on virus inactivation.

### Viability of viruses in droplets

The viability of viruses in droplets was studied in an environmental chamber (5518; Electro-Tech Systems) at room temperature (22 ± 1°C). For each virus suspension, droplets were exposed at low, intermediate, and high RH levels of 20%, 50%, and 80%, respectively. The targeted RH inside the environmental chamber was achieved by vaporizing ultrapure water with a humidifier or passing air through a desiccator. Fifteen minutes after the RH reached equilibrium, ten separate 1-μL droplets of virus suspension were spotted on a 6-well, polystyrene cell culture plate (SIAL0516; Sigma) with a 0.1-10-μL pipette. Droplets were incubated for 1 h, after which viruses were collected in 500 μL of LB medium by pipetting up and down several times. Samples were stored at -80°C immediately after collection until they were quantified by plaque assay. Control samples containing 10 μL of virus suspension in a sealed 1.5-mL microcentrifuge tube were incubated inside the environmental chamber during each experiment and collected in 500 μL of culture medium after 1 h.

### Plaque assay and relative viability

Virus samples were quantified by plaque assay as described previously [12]. Briefly, 10-fold serial dilutions of the collected samples were prepared. Fifty microliters of the serial dilutions, 450 μL of liquid culture of bacterial host, and 4.5 mL of soft agar were mixed and poured over agar plates. Plates were incubated at the bacterial host’s growth temperature for 24 h. The number of plaques on plates was counted, and the virus concentration in the samples were calculated as shown in Eq 1.

$$Viral\;titer\;(PFU/mL)\;=\;\frac{\#\;of\;plaques}{dilution\;factor\;*\;volume\;of\;virus\;suspension\;added\;to\;the\;plate}$$ (1)

The change in infectious viral concentration after 1 h of exposure was expressed as relative viability. Relative viability was calculated as the ratio of the post-exposure viral concentration, *C*_*post-exposure*_, to the pre-exposure concentration, *C*_*pre-exposure*_, as shown in Eq 2.

$$Relative\;Viability=\;\frac{C_{post-exposure}}{C_{pre-exposure}}$$ (2)

### Droplet evaporation rate

The droplet evaporation rate was determined as described previously [13]. Briefly, a microbalance (MSE3.6P; Sartorius) was placed inside an environmental chamber to weigh ten 1-μL droplets, which were spotted on a microscope cover glass (12-545-M; Fisher Scientific), over 1 h. Droplet mass was recorded at 1-min intervals. Droplet evaporation rate was calculated as shown in Eq 3.

$$Droplet\;evaporation\;rate=\frac{dm}{dt}=m_{t_{n}}-m_{t_{n-1}}$$ (3)

Where *m* is the mass of droplets (mg), and *t*_*n*_ is *n* min after droplets were spotted.

### Statistical analysis

All experiments were performed in triplicate. The relative viability of viruses was expressed as mean ± standard deviation. One-way ANOVA and a post-hoc analysis with Tukey’s HSD test were performed to determine significant differences (*P* < 0.05) in the relative viability of bacteriophages among different levels of media composition and among RH levels, respectively. Two-way ANOVA was performed to determine the interaction effect between media composition and RH. One-way ANOVA was performed to determine significant differences (*P*<0.05) in the evaporation rate of droplets with different initial solute concentrations.

## Results

### Salt

**Fig 2:**
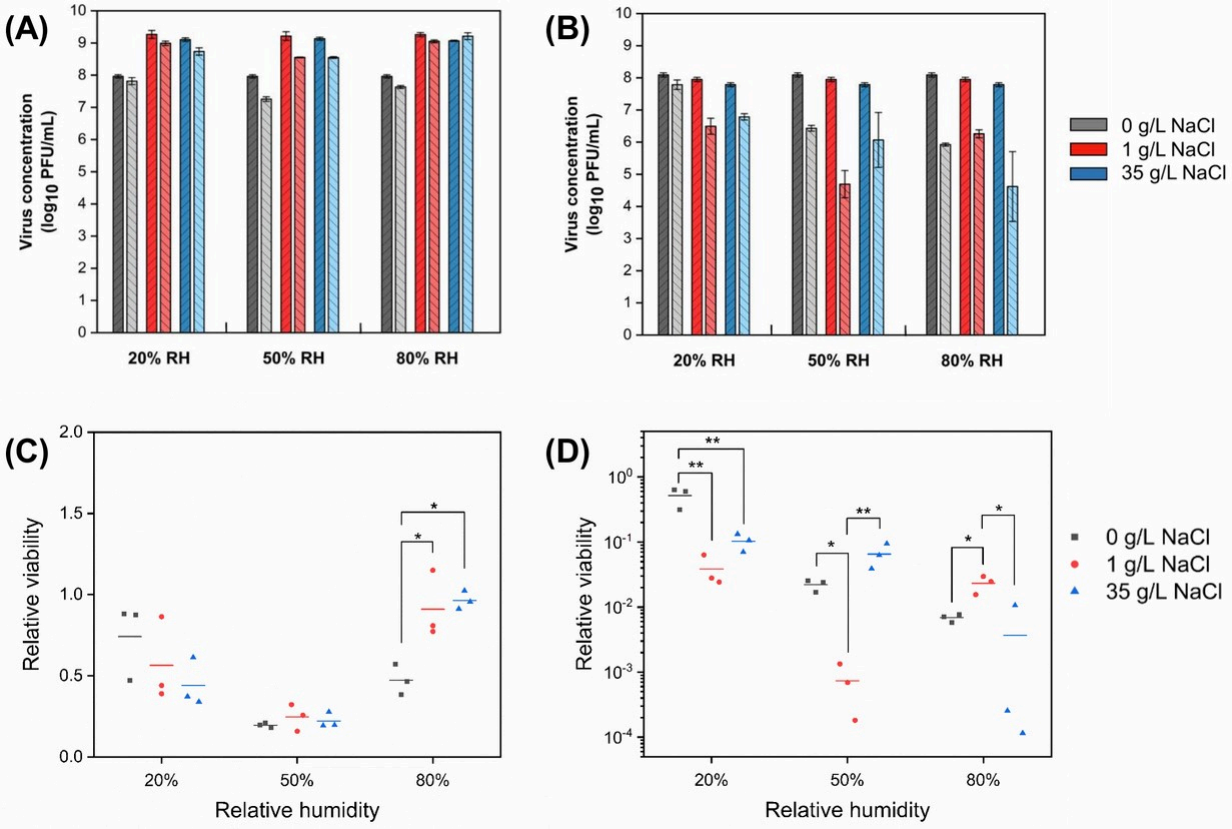
Concentration of bacteriophages (A) MS2 and (B) Φ6 in droplets with different initial sodium chloride concentration before (dark bars) and after (light bars) 1 h exposure to low, intermediate, and high RH (mean ± s.d. of triplicates). Relative viability of (C) MS2 and (D) Φ6 after 1 h exposure (lines shown the mean of triplicates). The number of virions in droplets at the start of the exposure experiments was 10^5^−10^6^ PFU.

The viability of MS2, a non-enveloped virus, and Φ6, an enveloped virus, was examined in droplets of different compositions at RHs of 20%, 50%, and 80% by plaque assay. As shown in Fig 2A and 2C, the effect of sodium chloride on MS2 viability was RH-dependent. MS2 decayed more in 35 g/L NaCl droplets than in 0 and 1 g/L NaCl droplets at 20% RH, while the pattern was opposite at 80% RH. At 80% RH, the viability of MS2 was significantly higher in droplets containing NaCl than in those without it, suggesting that NaCl had a protective effect at this RH condition. At 50% RH, the relative viability of MS2 was lower than at the other RHs and was similar across all NaCl levels.

The enveloped virus, Φ6, was generally more susceptible than the non-enveloped one, MS2. Thus, the relative viability of Φ6 is shown on a log scale, whereas that of MS2 is shown on a linear scale. The relative viability of Φ6 was less than 10% in droplets containing NaCl at all RHs after 1 h (Fig 2B and 2D). At 20% RH, the relative viability was significantly lower in droplets containing NaCl, at concentrations of both 1 and 35 g/L compared to 0 g/L. At 50% RH, the relative viability was lowest in 1 g/L NaCl droplets, while it was significantly higher in droplets containing 0 and 35 g/L NaCl. At 80% RH, the relative viability was significantly higher in droplets containing 1 g/L NaCl compared to 35 g/L NaCl.

### pH

**Fig 3:**
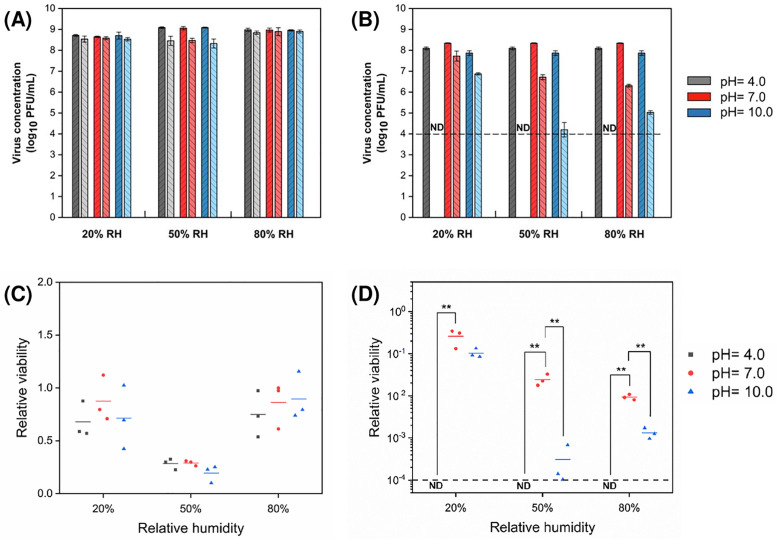
Concentration of bacteriophages (A) MS2 and (B) Φ6 in droplets with different initial pH values before (dark bars) and after (light bars) 1 h exposure to low, intermediate, and high RH (mean ± s.d. of triplicates). Relative viability of (C) MS2 and (D) Φ6 after 1 h exposure (lines show the mean of triplicates). The number of virions in droplets at the start of the exposure experiments was 10^5^−10^6^ PFU. The dark gray dashed line indicates the detection limit (10^4^ PFU/mL) of plaque assay. ND indicates no viable virus was detected.

The relative viability of MS2 in droplets at pH values of 4.0, 7.0, and 10.0 (i.e., acidic, pH-neutral, and basic) was generally similar at any one RH level (Fig 3A and 3C). At 20% RH, MS2 survived better, although not significantly, in pH-neutral droplets than in more acidic or more basic droplets. There were no significant differences in viability across pH at the other two RHs. Regardless of pH, viability was significantly lower at 50% RH compared to the other RHs.

The relative viability of Φ6 differed significantly by pH, while the patterns in viability were similar across all three RHs (Fig 3B and 3D). At a pH of 4.0, no viable Φ6 was detected in either the control solution or the evaporating droplets after 1 h, suggesting a strong inactivation effect of acidic conditions on Φ6. At a pH of 10.0, the virus decayed by ~1–3 log_10_ units depending on RH, while the virus survived best in pH-neutral droplets (7.0), in which it decayed by ~1–2 log_10_ units depending on RH. At both these pHs, relative viability was greater at 20% RH compared to the two higher RHs.

### Protein

**Fig 4:**
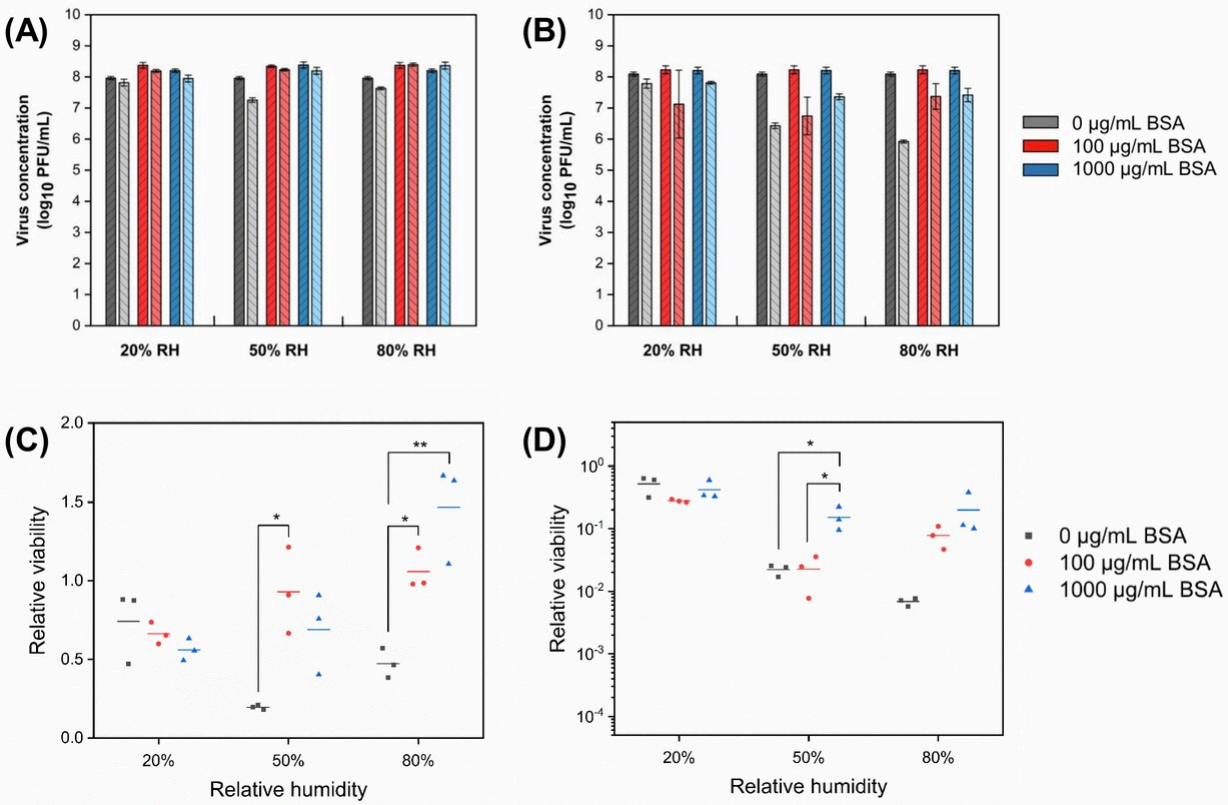
Concentration of bacteriophages (A) MS2 and (B) Φ6 in droplets with different initial protein concentration before (dark bars) and after (light bars) 1 h exposure to low, intermediate, and high RH (mean ± s.d. of triplicates). Relative viability of (C) MS2 and (D) Φ6 after 1 h exposure (lines show the mean of triplicates). The number of virions in droplets at the start of the exposure experiments was 10^5^−10^6^ PFU.

Similar to salt, the effect of protein on MS2 was also RH-dependent (Fig 4A and 4C). The relative viability of MS2 decreased as the concentration of BSA increased in droplets at 20% RH. However, at RHs of 50% and 80%, the relative viability was higher in the presence of BSA. At 50% RH, the viability of MS2 was reduced by only 7% in droplets containing 100 μg/mL BSA after 1 h, a significantly lower loss than the >80% reduction in droplets that did not contain any BSA. At 80% RH, there was no decay in droplets containing BSA, regardless of its concentration, suggesting that BSA has a protective effect on the viability of MS2.

Similar to its effect on MS2, BSA protected Φ6 from inactivation in droplets at intermediate and high RHs (Fig 4B and 4D). At 20% RH, the relative viability of Φ6 was similar in droplets with and without BSA. However, at 50% RH, the relative viability of Φ6 was significantly higher in droplets containing 1000 μg/mL BSA than in droplets with 0 or 100 μg/mL BSA. At 80% RH, the presence of BSA, regardless of its concentration, reduced the decay of Φ6 in droplets, suggesting its protective effect on viruses in droplets again.

### Surfactant

**Fig 5:**
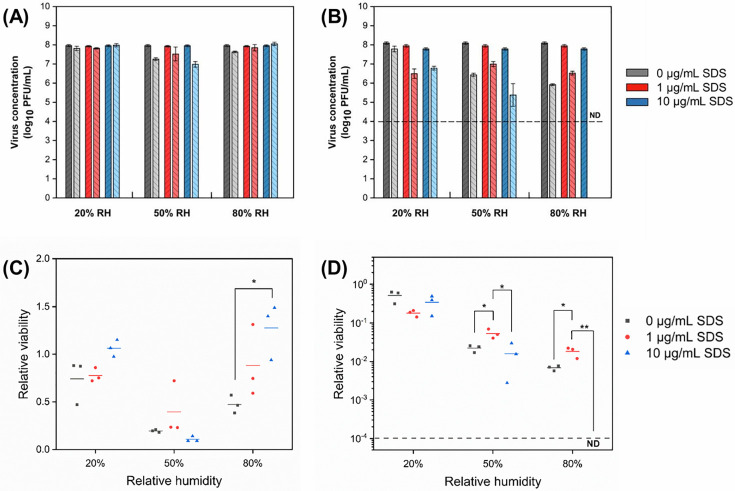
Concentration of bacteriophages (A) MS2 and (B) Φ6 in droplets with different initial surfactant concentration before (dark bars) and after (light bars) 1 h exposure to low, intermediate, and high RH (mean ± s.d. of triplicates). Relative viability of (C) MS2 and (D) Φ6 after 1 h exposure (lines show the mean of triplicates). The number of virions in droplets at the start of the exposure experiments was 10^5^−10^6^ PFU. The dark gray dashed line indicates the detection limit (10^4^ PFU/mL) of plaque assay. ND indicates no viable virus was detected.

The relative viability of MS2 in droplets with different surfactant concentrations is shown in Fig 5A and 5C. MS2 generally survived better when SDS was present in droplets, and relative viability increased with SDS concentration at 20% and 80% RH. MS2 incurred no decay in droplets containing 10 μg/mL SDS, whereas it at least lost 25% viability in droplets containing no SDS. The relationship between viability and SDS concentration differed at 50% RH, at which MS2 survived best in droplets with 1 μg/mL SDS, but decayed most in droplets containing 10 μg/mL SDS. As shown in Fig 5B and 5D, SDS did not significantly affect the viability of Φ6 in droplets at RHs of 20%. However, 10 μg/mL SDS induced a significantly higher inactivation of Φ6 at RHs of 50% and 80%; no viable Φ6 was recovered from droplets containing 10 μg/mL SDS at high RH after 1 h.

### Relative humidity

U-shaped patterns in the viability of MS2 against RH were observed in droplets composed of salt and surfactant, as well as in pH-adjusted droplets. Specifically, the relative viability of MS2 was lowest at 50% RH, while it was significantly higher at RHs of 20 and 80%. A different pattern was observed for the viability of MS2 in droplets containing protein, in which the relative viability of MS2 generally increased as RH increased. Two-way ANOVA indicated that there was a main effect of RH, but not salt or pH, on the viability of MS2 in droplets. In droplets composed of protein and surfactant, both RH and droplet composition (i.e., protein and surfactant) had a statistically significant effect on the relative viability of MS2. Meanwhile, there was an interaction effect between RH and droplet composition on the viability of MS2 in droplets containing salt, protein, and surfactant, but not between RH and the pH of droplet media.

The effect of RH on the viability of Φ6 was different from that on MS2. Instead of following a U-shaped pattern, the viability of Φ6 generally decreased as RH increased in droplets containing salt and surfactant, and in pH-adjusted droplets. The pattern was slightly different for droplets containing protein due to the protective effect from BSA, which made the trend more U-shaped. Statistical analysis suggests that there was a main effect of RH on the viability of Φ6 in droplets composed of surfactant. There was an interaction effect between RH and salt, protein, and pH on the survival of Φ6, respectively.

## Discussion

Our results show that the chemistry of carrier droplets has significant impacts on the viability of both non-enveloped and enveloped viruses. The results suggest that the chemical composition of carrier droplets can influence the stability of viruses when they are released into the environment. While salt, pH, and surfactant reduced the viability of viruses at most RH conditions, protein provided some protection against virus decay in droplets. The effect of chemical composition was coupled with RH, which emphasizes the importance of exploring the effects of droplets’ chemical composition and environmental factors simultaneously in investigating the survival of viruses in the environment.

### Salt

Sodium chloride promoted the inactivation of viruses at low RH, but it did not affect, and sometimes even reduced, the decay of viruses at intermediate and high RHs. These seemingly conflicting results can be explained by two distinct mechanisms. Firstly, previous studies have reported that NaCl inactivates viruses, possibly by damaging viral RNA [48, 49], although the mechanism of inactivation has not been explicitly identified [48, 50]. Our results confirmed the effect, as decay of MS2 and Φ6 was enhanced in droplets containing NaCl at 20% RH. On the other hand, studies have suggested that viruses tend to aggregate in solutions with high salt concentration [51–54]. The formation of large virus aggregates increases virus stability in such environments [52]. Although other studies have demonstrated that low levels of salt (e.g., initial concentration of 1 g/L NaCl in our droplets) do not effectively facilitate the formation of virus aggregates [55, 56], we speculate that virus aggregation would be enhanced in evaporating droplets. This is because the salt concentration increases in evaporating droplets as they lose water, especially when they are close to desiccation, while the decrease in droplet volume brings viruses into contact with one another. We hypothesize that the increased relative viability of MS2 and Φ6 in droplets containing sodium chloride at 80% RH is due to the formation of virus aggregates. Further studies are needed to provide direct evidence supporting this hypothesis.

**Fig 6:**
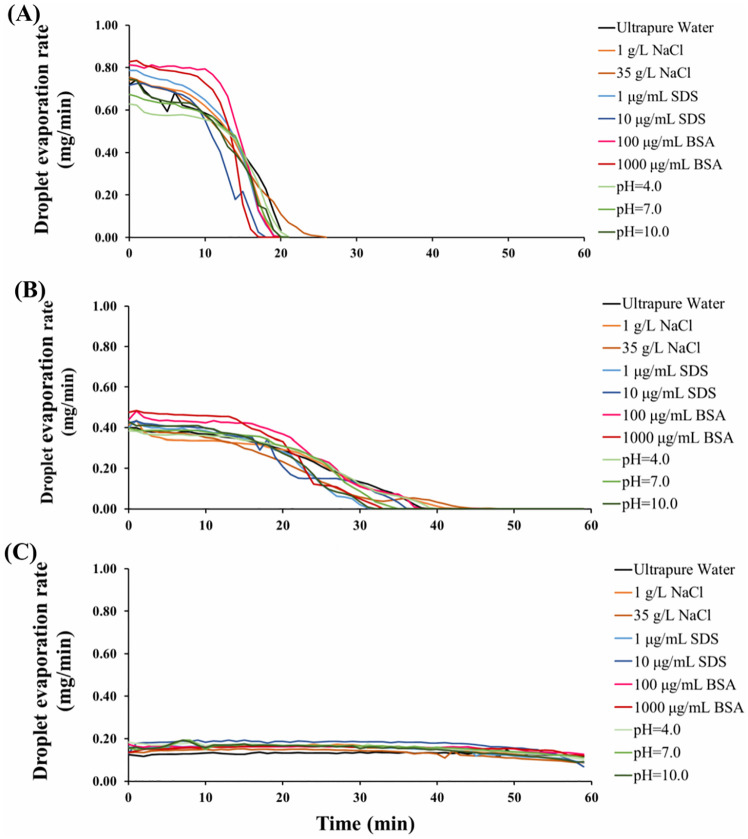
Evaporation rate of droplets with different chemical compositions at (A) 20% RH, (B) 50% RH, and (C) 80% RH. The lines represent the mean of duplicates. Error bars are not shown to facilitate visualization. The relative standard deviation averaged 7%, 18%, and 7% at 20%, 50%, and 80% RH, respectively.

The observed RH-dependent effect of salt on the viability of viruses suggests that the relative contribution of the abovementioned mechanisms may vary at different RH conditions. The evaporation kinetics of droplets at various RHs seems to play an important role. Droplets with an initial volume of 1 μL evaporate rapidly at low RH (Fig 6A), desiccating within 15 minutes at 20% RH, whereas the evaporation process is much slower at high RH (Fig 6B and 6C). It is plausible that at low RH, droplets quickly desiccate before considerable amounts of virus aggregates are generated, in which case the inactivation effect of NaCl dominates and results in enhanced decay of viruses. Conversely, at high RH, droplet evaporation is much slower, allowing viruses to form aggregates and thus protecting viruses from inactivation. Again, additional investigation is needed to test this hypothesis.

### pH

Our results demonstrate that pH affects the stability of MS2 and Φ6 differently in droplets. MS2 survived equally well in acidic, pH-neutral, and basic droplets, whereas Φ6 survived best in pH-neutral droplets and decayed more in acidic or basic droplets. Previous studies have reported that viruses in bulk solutions are sensitive to pH [57, 58]. Both non-enveloped and enveloped viruses are generally more susceptible in acidic and basic solutions than in pH-neutral solutions [57]. At extreme pHs, viruses decay due to the denaturing of surface proteins and the hydrolysis of the viral genome [48, 59]. However, MS2 appears to be insensitive to pH. In a previous study, a moderate decay rate of ~0.5 log_10_ unit per day was observed for MS2 in bulk solutions at pH values of 4 and 10, and MS2 retained its viability when the solution was pH-neutral. The effect of pH on the viability of enveloped viruses is generally more noticeable than its effect on non-enveloped viruses [57], consistent with our observation of pronounced decay of Φ6 in acidic solutions across all RH levels. Besides the protein denaturing effect, the fusion of enveloped viruses’ membrane structure caused by extreme pH also leads to inactivation [48]. Low-pH treatment is widely used in monoclonal antibody purification processes to inactivate viruses because of its reliable performance (e.g., > 4 log_10_ decay) on enveloped viruses [60].

In addition to the inactivation effect induced by pH, the dynamic change in pH of evaporating droplets can also affect virus survival. Although the pH of all virus suspensions was adjusted to the target pH at the beginning of experiments, the pH is likely to change as droplets evaporate. The loss of water will enrich ions, such as H_3_O^+^ and OH^-^, which may create pH-gradients inside droplets [46]. Additionally, since droplets were exposed to ambient air, the uptake of CO_2_ and formation of carbonic acid may lower the pH of droplets, but determining the extent of this process in evaporating droplets is challenging. Therefore, the pH of droplets is not expected to remain constant at its initial value throughout the experiment. The dynamic change in the pH of evaporating droplets introduces uncertainties in understanding its effect on the survival of viruses. Tools to monitor the real-time pH in evaporating droplets are necessary to fully explain the effect of pH on the viability of viruses in this complex system.

### Protein

The relative viability of MS2 and Φ6 was elevated in droplets containing BSA at RHs of 50% and 80%. Previous studies have found that the decay of viruses was greatly reduced in both aerosols and droplets supplemented with human respiratory fluid or fetal calf serum [17, 18]; protein may provide a protective effect. For example, influenza virus retained its viability in aerosols across a wide range of RHs after 1 h when the aerosolization media was supplemented with extracellular matrix from human bronchial epithelial cells. Here, we suspended viruses in media containing BSA and observed a similar protective effect. The detailed mechanism by which proteins protect viruses from decay remains unknown. Researchers have proposed that the inactivation of viruses in aerosols and droplets mainly happens at the air-water interface [61, 62]. The presence of proteins in droplets may reduce the solution surface tension, which inhibits viruses from reaching the air-water interface [63, 64]. Another possible mechanism is that potentially damaging compounds may first act on free proteins in droplets instead of those on the surfaces of viruses. Quantitative information on residual “free protein” in droplets over the course of exposure would be useful to test this hypothesis. It is also possible that proteins in solution may interact with those on the surface of viruses and help stabilize them.

### Surfactant

Surfactants have been reported previously to enhance the inactivation of viruses [65, 66], which is in agreement with our results for Φ6. High concentrations of surfactant are very effective in inactivating enveloped viruses. For example, > 4 log_10_ reduction has been reported after 1 h incubation in an 80 μM surfactin solution [67]. According to electron microscopy, the decay mechanism was concluded to be the disintegration of the lipid membrane and partial disintegration of the protein capsid on enveloped viruses. Since the initial concentrations of SDS in our droplets were much lower (3.4 and 34 μM), the magnitude of virus decay in our study was lower than previously reported.

Since a lipid membrane is present only in enveloped viruses, the effect of surfactant on non-enveloped viruses is much weaker than on enveloped viruses [67]. Interestingly, we observed less decay of MS2 in droplets containing SDS compared to those without, suggesting a protective effect of SDS on the survival of non-enveloped viruses in droplets. Surfactants could protect viruses in a similar manner as proteins. Surfactants are known to strongly affect the surface tension of solutions, especially when the surfactant concentration is below the critical micelle concentration, beyond which micelles start to form and the surface tension of solutions remains relatively constant. Since the concentration of SDS examined in our study is much lower than its critical micelle concentration (8.2 mM), the presence of SDS in droplets could affect the surface tension and protect viruses from decay by hampering their ability to reach the air-water interface.

To our best knowledge, the effect of the chemical composition of droplets on the transmission of human or mammalian viruses has not been reported previously. Our results with model bacteriophages indicate that their survival in droplets is sensitive to the concentrations of different components in the droplets. This observation agrees with the findings from a previous study that investigated the effects of salt and protein on the survival of influenza virus in droplets; high salt concentrations were correlated with greater virus inactivation while protein protected the virus [17]. Since viruses must retain their infectivity to transmit successfully, our results imply that the chemical composition of droplets may influence virus transmission by modulating the survival of viruses.

### Relative humidity

An association between RH and virus transmission has been reported. For example, the incidence of influenza A in Hong Kong increased with higher RH, and the number of positive test results for influenza A was negatively correlated with RH in Singapore [68, 69]. Results from these epidemiological studies suggest that RH affects virus transmission. Furthermore, studies have demonstrated the effect of RH on the survival of human viruses (e.g., SARS-CoV-2 [70, 71]), underlining the importance of understanding the effect of RH on virus transmission.

RH has large impacts on the viability of viruses in droplets, larger than the effect of chemical composition in some cases. We observed U-shaped patterns in the viability of MS2 against RH, and monotonically decreasing relationships between the viability of Φ6 and RH, respectively, in droplets of different compositions. We reported previously that the viability of MS2 and Φ6 in droplets composed of culture medium follows U-shaped patterns, in which the lowest viability occurs at 55% and 85% RH, respectively [13]. Many other studies have reported a similar pattern with greater decay at intermediate RHs than at lower or higher RHs [17, 18, 25, 27, 72]. The viability patterns observed in this study for Φ6, decreasing with RH rather than U-shaped, seem to conflict with results in the literature. However, we examined the viability of Φ6 between 20% and 80% RH in the current study. Over this range, the viability of viruses also decreased monotonically in previous studies; we have shown that the minimum viability of Φ6 in droplets occurs around 85% RH, beyond the range examined in the present study [13].

As we concluded in our prior study [13], RH affects the viruses’ viability mainly by controlling droplet evaporation kinetics, inducing changes in solute concentrations and the cumulative dose of harmful compounds to which viruses are exposed. At intermediate RH, the cumulative dose is higher because the solute concentrations increase relatively quickly and are then maintained at a high level throughout the experiment. While our previous work focused on viruses in their prescribed culture medium, results of the present study indicate that their viability follows the same pattern in droplets consisting of culture medium diluted 100x in ultrapure water and lacking salt, protein, and surfactant. Components in LB medium that are potentially harmful for viruses, though diluted, can accumulate as droplets evaporate and eventually cause virus inactivation over time.

RH is the major factor that determines droplet evaporation kinetics, as shown in Fig 6. The initial evaporation rate was much higher at 20% RH than at 50% and 80%. At 20% and 50% RH, droplets fully desiccated in 1 h. At both conditions, the evaporation rates were relatively steady at the beginning (the first ~10 min and ~15 min for 20% and 50% RH, respectively), but later gradually decreased. However, at 80% RH the evaporation rate was more consistent throughout the experiment, and droplets did not fully evaporate within 1 h.

Besides ambient RH, droplet composition can affect evaporation rates as well. At certain RH conditions, the evaporation kinetics varied with chemical composition and initial solute concentration. Droplets containing BSA generally evaporated faster than droplets containing other components at 20% and 50% RH. Droplets containing 1 g/L NaCl evaporated faster than those containing 35 g/L NaCl. A previous study demonstrated that the evaporation rates of droplets containing less than 5.8 g/L NaCl was almost two times higher than for droplets containing 58 g/L NaCl at RH < 60% [73]. The authors concluded that Marangoni flows induced by surface tension gradients, which originated from local peripheral salt enrichment, caused the difference in evaporation rate. We observed that droplets with higher initial SDS concentration evaporated slower than those with lower initial SDS concentration at RHs of 20% and 50%. However, the result was completely the opposite at 80% RH, at which droplets containing 10 μg/mL SDS evaporated significantly faster than those containing 1 μg/mL SDS. Droplets containing different initial BSA concentrations and at different initial pH values had similar evaporation rates across all RH levels. Since the evaporation kinetics determine the change in solute concentrations and cumulative dose, it is necessary to understand the influence of droplet chemical composition and concentrations on the evaporation rates of virus-containing droplets.

### Limitations

While this study provides novel results on the viability of viruses in evaporating droplets of different compositions over a range of RHs, it does not examine how the surface material upon which viruses are deposited might affect viability. Previous studies have reported that the persistence of viruses in droplets depends on the type of material (e.g., plastic vs. steel) [74]. It is possible that material exchange between surfaces and droplets (e.g., dissolution of metal ions into droplets) leads to accumulation of surface materials in droplets and inactivates viruses. It will be interesting to investigate the effects of the interplay among surface materials, droplet composition, and environment on the survival of viruses in droplets. Additionally, we have focused on the biological inactivation of viruses in droplets but not on their physical behavior, which likely depends on physicochemical characteristics of the droplets. Future studies should be conducted in this area, although pinpointing viruses within droplets is challenging. Results might help explain the protective or inactivating effect of certain media components we observed in this study. Previous studies have demonstrated differences in the persistence of viruses on surfaces as a function of initial viral titer in the inoculum [75, 76]. Investigating the role of viral titer, which might affect aggregation and other characteristics, on virus survival is another interesting research question. Lastly, this study reported findings from two bacteriophage models, which may not fully represent human viruses. Human viruses, such as influenza virus and coronavirus, should be used in future studies to elucidate the effects of droplets’ chemical composition and RH on virus survival and transmission of viruses.

To conclude, we demonstrated that both the chemical composition of droplets and RH strongly affect the viability of non-enveloped and enveloped viruses. The effects of sodium chloride and SDS varied by RH level and virus type. pH did not affect the viability of MS2 but effectively inactivated Φ6 in solutions at pHs of 4 and 10. BSA generally preserved the viability of MS2 and Φ6 in droplets. We also found that the viability of viruses in droplets of certain compositions was RH-dependent at most conditions. Our results reveal that two factors contribute to the inactivation of viruses in droplets: (1) droplet evaporation kinetics, which are controlled by RH; and (2) inactivation or protective effects induced by chemicals. Additionally, the physical behavior of viruses, such as forming aggregates and partitioning to the air-liquid interface, resulting from changes in droplets’ characteristics may also affect inactivation. Results from our study are meaningful in predicting the persistence of viruses in droplets of various compositions in the environment and infectious disease transmission.

## Supporting information

S1 File Raw data that is the basis for the Figs 2–5 in the manuscript. (XLSX)

S2 File Raw data that is the basis for the Fig 6 in the manuscript. (XLSX)
